# The Impact of US Food and Drug Administration’s Advisory and Enforcement Actions on US Sales of Esco Bars Products in 2023: Synthetic Control Approach

**DOI:** 10.2196/81033

**Published:** 2026-03-12

**Authors:** Hyungsik Shin, Jonathan Hannings, Zachary Cahn, Xin Xu, Robin Toblin, Matthew Farrelly, Brian A King

**Affiliations:** 1 Center for Tobacco Products, US Food and Drug Administration, 10903 New Hampshire Avenue, Silver Spring, MD, 20993, United States, 1 (888) 463-6332

**Keywords:** unauthorized e-cigarettes, illicit sales of e-cigarettes, US Food and Drug Administration, FDA, FDA advisory and enforcement actions, synthetic control method, SCM, difference-in-differences, DiD

## Abstract

**Background:**

In 2023, Esco Bars was the second most commonly reported e-cigarette brand used among US middle and high school student e-cigarette users. These products have not been authorized for sale in the United States by the US Food and Drug Administration (FDA). On May 12, 2025, and May 25, 2023, the FDA issued an import alert and a warning letter, respectively, to the manufacturer requiring them to immediately remove these unauthorized products from the market. On June 22, 2023, and July 27, 2023, the FDA also issued warning letters to US retailers and distributors, respectively, regarding the illicit sale of these unauthorized products. This study evaluated the impact of these advisory and enforcement actions on retail sales of Esco Bars in the United States.

**Objective:**

We evaluated the impact of these 2023 FDA advisory and enforcement actions on illicit sales of unauthorized Esco Bars to better inform future regulatory decision-making.

**Methods:**

This study used a synthetic control method to evaluate the impact of 2023 FDA advisory and enforcement actions on Esco Bars sales based on weekly sales data from the NielsenIQ Retail Measurement Service between January 1, 2023, and December 30, 2023. Data came from the 48 contiguous states and were not available for internet and vape shop sales. First, we standardized the data by transforming the weekly sales into *z* scores. Second, we created a synthetic comparison for Esco Bars sales based on sales of other e-cigarettes for which the FDA did not take action during the analytical period. Finally, we calculated the difference in sales between Esco Bars and the comparison group before and after the FDA actions. To assess the robustness of the findings, we used other standardization methods for sensitivity analyses.

**Results:**

By the last week of 2023, actual Esco Bars’ weekly sales were 68.5% lower than the estimated sales in the comparison group (*P*=.02). Over the 5-month period following FDA advisory and enforcement actions, Esco Bars product sales were reduced by approximately 1.7 million equivalized units (*P*=.06). These findings were robust across sensitivity analyses.

**Conclusions:**

The FDA’s advisory and enforcement actions substantially reduced sales of Esco Bars products in the United States, with a sustained impact noted over a 5-month period. These findings underscore the importance and impact of FDA actions against unauthorized tobacco products as part of a comprehensive regulatory approach.

## Introduction

E-cigarettes have been the most commonly used tobacco product among youth in the United States since 2014, surpassing conventional cigarettes [1]. E-cigarettes can generally have a lower risk than conventional cigarettes and have the potential to benefit adult smokers who transition completely [2]. However, youth uptake and use of these products is problematic, including because e-cigarettes contain nicotine, which is a highly addictive chemical compound. Nicotine exposure can be associated with multiple adverse health consequences, including harming adolescent brain development and priming the brain for addiction to other drugs [3-5].

Youth e-cigarette use prevalence increased rapidly in the early 2010s and peaked in 2019 at nearly 28%, largely driven by flavored cartridge-based systems [6]. Since then, the market landscape has continued to evolve, and flavored disposable products have become the most popular type of e-cigarettes among youth [7]. According to the 2023 National Youth Tobacco Survey, approximately 60.7% of youth reporting current e-cigarette use used disposable e-cigarettes, and Esco Bars was the second most commonly reported brand used among youth who currently use e-cigarettes [7].

In the United States, tobacco products not on the market prior to 2007, including e-cigarettes, are required to submit a premarket tobacco product application (PMTA) and receive authorization by the US Food and Drug Administration (FDA) before the product can be marketed. The manufacturer of Esco Bars submitted a PMTA. However, in 2022, the FDA issued a refuse to accept (RTA) letter to the manufacturer of Esco Bars, finding that its submitted premarket tobacco product application did not provide sufficient information to permit substantive review per regulatory requirements. Receipt of an RTA letter indicates that a manufacturer does not have authorization to legally market its products in the United States. Nevertheless, the manufacturer continued to produce and sell these products.

As a result of common youth use of Esco Bars, and following the manufacturer’s lack of compliance with regulatory review decisions, the FDA took targeted advisory and enforcement actions as a part of a comprehensive approach to reduce youth use of tobacco products [8]. The FDA can issue untitled letters and warning letters as advisory actions to encourage voluntary compliance when responding to illicit tobacco product marketing. Warning letters provide instructions to a company (ie, manufacturer, distributor, or retailer) in violation of FDA regulations, including directions for prompt action to address the violations and a timeline for a response [9]. FDA may also pursue enforcement measures including import alerts, civil money penalties, seizures, and injunctions [10,11]. Import alerts signal that violative products may be detained upon entry into the country [12].

To underscore the FDA’s commitment to protecting youth against unauthorized e-cigarettes, the FDA issued an import alert for Esco Bars products on May 12, 2023 [13]. Additionally, the FDA issued a warning letter to the manufacturer of Esco Bars products on May 25, 2023, as the firm had been manufacturing, distributing, and/or importing these unauthorized tobacco products in the United States despite prohibitions stated in the RTA letter [14]. Following these actions, FDA further issued warning letters to retailers and distributors of e-cigarette products on June 22, 2023, and July 27, 2023, respectively [15,16], for selling and/or distributing unauthorized Esco Bars products.

Existing studies have analyzed FDA warning letters on e-cigarettes issued to retailers and manufacturers between 2018 and 2021 by violation type and company characteristic [17,18], including one study that also assessed compliance among online retailers [17]. However, none of these studies have examined the impact of these advisory actions on e-cigarette sales. To our knowledge, this is the first study evaluating the impact of FDA advisory and enforcement actions on illicit tobacco product sales following noncompliance with regulatory review decisions. To address this gap in the scientific literature, this study explores the association between the FDA’s targeted advisory and enforcement actions in 2023 and subsequent changes in Esco Bars product sales by the end of 2023.

## Methods

### Overview

#### Data

The FDA’s Center for Tobacco Products licenses NielsenIQ Retail Measurement Service (RMS) data, which includes weekly tobacco product sales from 48 contiguous states (not including Hawaii and Alaska). NielsenIQ RMS data come from several sources, including electronic point-of-sale data from stores through product bar code checkout scanners at retailer registers, coding of retail circulars (eg, in-store flyers and advertisements promoting products), and in-store audits (ie, field auditors who capture in-store display information promoting products). NielsenIQ uses proprietary statistical methods to project nationally representative sales of nicotine and tobacco products by universal product code.

#### Unit Sales

The FDA’s Center for Tobacco Products receives NielsenIQ tobacco product sales data in both raw and equivalized units. The equivalized unit was created by NielsenIQ to provide a standardized volume measure for products with varying package sizes. Equivalized unit can vary by tobacco product type. For e-cigarettes, 1 equivalized unit is equal to 1 disposable device, prefilled pod, starter kit, or e-liquid bottle. For this study, we used weekly e-cigarette equivalized unit sales from January 1, 2023, to December 30, 2023.

#### Retail Channel

Weekly e-cigarette equivalized sales were assessed by combining two primary NielsenIQ retail channels: (1) convenience stores, which encompass smaller stores with a limited selection of grocery products, including chain, franchise, and independent convenience stores; and (2) Extended All Outlets Combined, which is a combination of food and drug stores plus select mass merchandiser (eg, Target), club store (eg, Sam’s Club), military commissary (eg, Army & Air Force Exchange Service), and dollar store (eg, Dollar General) accounts [19].

#### Sample

NielsenIQ reports e-cigarette brands as listed on external product packages. We identified 4 Esco Bars-related sub-brands in NielsenIQ RMS data in 2023, including Esco Bars, Esco Bars Carsonator, Esco Bars Fruitia, and Esco Bars Ripe Collection. According to the data, these 4 sub-brands are produced by the same manufacturer and share the major brand, Esco Bars, in their names. Therefore, we reported all 4 sub-brands as Esco Bars products for the purposes of this study. Following the same approach, we combined other sub-brands that are produced by the same manufacturers and share similar major brand names. Details about these sub- and major brands are available upon request. We included only brands that provided full sales data for the entire study period. In addition, we excluded e-cigarette products that do not contain nicotine, that is, accessories (eg, e-cigarette battery, coils, and carrying cases), as they are not applicable comparisons for Esco Bars for this study. These excluded products represented very few e-cigarette equivalized unit sales (<0.1%) during the analytical timeframe.

### Study Design

We used the synthetic control method (SCM) to draw inferences about the likelihood that the intervention had a causal effect on Esco Bars’ sales. This is similar to a conventional difference-in-difference (DiD) approach, as both methods compare changes over time between a group exposed to the intervention and an unexposed comparison group [20]. Although DiD models have been widely used to assess potential effects of policy interventions, their validity critically relies on the parallel trends assumption (ie, that the treatment and control groups would have changed in the same way over time if the intervention had not occurred, and thus changes during the postintervention period are due to the treatment itself) [21]. DiD was not suitable for this study because all potential comparison e-cigarette brands failed parallel trends tests. Therefore, we used the SCM, which provides a systematic way of creating an appropriate comparison when the parallel trends assumption cannot be met [22].

In this study, the intervention group was Esco Bars, which was subject to FDA advisory and enforcement actions in 2023. The intervention period was between May 14, 2023, and July 29, 2023. Although the FDA first issued an import alert for Esco Bars products on May 12, 2023, we used May 14, 2023, as the first date of intervention because NielsenIQ weekly sales are tracked from Sunday through Saturday. May 12, 2023, was a Friday falling in the week ending May 13, 2023. For the same reason, we chose July 29, 2023, as the last day of the intervention because July 27, 2023, (the date when the FDA took the last advisory action, issuing warning letters to distributors, against Esco Bars product sales in 2023) was a Thursday falling in the week ending on July 29, 2023. We assumed that adjusting the intervention period by one or two days based on NielsenIQ weekly sales would not meaningfully affect our findings.

To be conservative and fully evaluate the combined impact of multiple FDA advisory and enforcement actions together, we defined the postintervention period as 5 months between July 30 and December 30, 2023, and reported our major findings accordingly. In Figure S1 in Multimedia Appendix 1, we present additional estimates, which include the impact of advisory and enforcement actions during the intervention period, starting from May 14, 2023.

The synthetic comparison of Esco Bars was generated from a set of e-cigarettes that did not encounter any FDA advisory and enforcement actions, including import alerts, warning letters, refuse to file letters, RTAs, or marketing denial orders during the analytical period.

Following these criteria, we identified a total of 51 e-cigarette brands for the “donor pool,” that is, all brands that were eligible to be part of the comparison, from the 2023 NielsenIQ RMS data. Among these 51 brands, weights for the synthetic comparison were optimized based on their closeness of fit to the actual Esco Bars’ sales during the preintervention period, that is, from January 1, 2023, to May 13, 2023. In the main model, the synthetic comparison consisted of a weighted average of 10 selected brands. Details about these brands and their weights are reported in Table S1 in Multimedia Appendix 1.

A proper execution of the SCM requires that variables in the data are rescaled to correct for differences in size between units [23]. Weekly unit sales varied substantially by brand in the final sample, ranging from less than 1000 equivalized units to a little more than 5 million equivalized units each week. To address the issue, following existing studies [24,25], we rescaled weekly equivalized unit sales using a *z* score standardization method. For a given week, we subtracted each brand’s preintervention averages from its weekly sales and divided the result by its pre-intervention standard deviation. In this case, a 1-unit change in standardized sales for a given brand is equivalent to a change of 1 preintervention standard deviation. To assess the sensitivity of our findings to the choice of standardization methods, we conducted sensitivity analyses using two alternatives: (1) min-max normalization—subtracting the preintervention minimum and dividing by the preintervention range, and (2) robust rescaling—subtracting the preintervention median and dividing by the preintervention interquartile range. A more detailed methodological approach for each standardization method is provided in Multimedia Appendix 1.

### Statistical Analyses

The application of conventional statistical approaches to assess significance is not available for the SCM approach [22,26]. Therefore, we estimated the *P* value using the concept of the Fisher exact test [27]. First, we calculated differences in sales between Esco Bars and its synthetic comparison during the postintervention period. This difference was calculated as the postintervention mean squared error (MSE). Then we calculated the ratio of the postintervention MSE to the preintervention MSE to determine the relative magnitude of the intervention effect. Second, we constructed synthetic estimates for every comparison brand and calculated intervention effects for each brand. These differences for the comparison brands are false intervention effects because these brands should not have been affected by the intervention (ie, FDA advisory and enforcement actions against illicit sales of Esco Bars). Then, we compared the intervention effect for Esco Bars to the false intervention effects of all 51 brands. Finally, we obtained a *P* value as the share of false intervention effects greater than or equal to the actual intervention effect [28]. If the ratio is sufficiently small, we can reasonably conclude that the intervention effect is unlikely to be a chance result. Technical details of the SCM and Fisher exact test are presented in Multimedia Appendix 1.

All data analyses were independently conducted using Stata version 16.1 (StataCorp LLC), R version 4.3.1 (R Foundation for Statistical Computing), and Python version 3.11.5 (Python Software Foundation) together with Anaconda version 23.10.0 (Anaconda, Inc).

### Ethical Considerations

We used deidentified aggregated retail sales data licensed from NielsenIQ for this study. All sales data were fully anonymized. Therefore, this study did not involve human participants, human tissue, or identifiable personal data, and ethical approval was not required in accordance with institutional and national guidelines.

## Results

### Findings

Of the 52 brands in our final sample, including Esco Bars, 67.3% (35/52) of brands represented only disposable e-cigarettes, 1.9% (1/52) represented only prefilled cartridges, and 30.8% (16/52) represented multiple types of e-cigarette products. The average weekly sales were estimated at approximately 114,000 equivalized units per brand (SD 662,000), with the median at 1400 units per week (IQR 10,000). The large difference between the mean and median reflects the disproportionate share of sales captured by the most popular brands in our sample, providing additional supporting evidence for using standardized sales prior to implementing the SCM.

**Figure 1.**
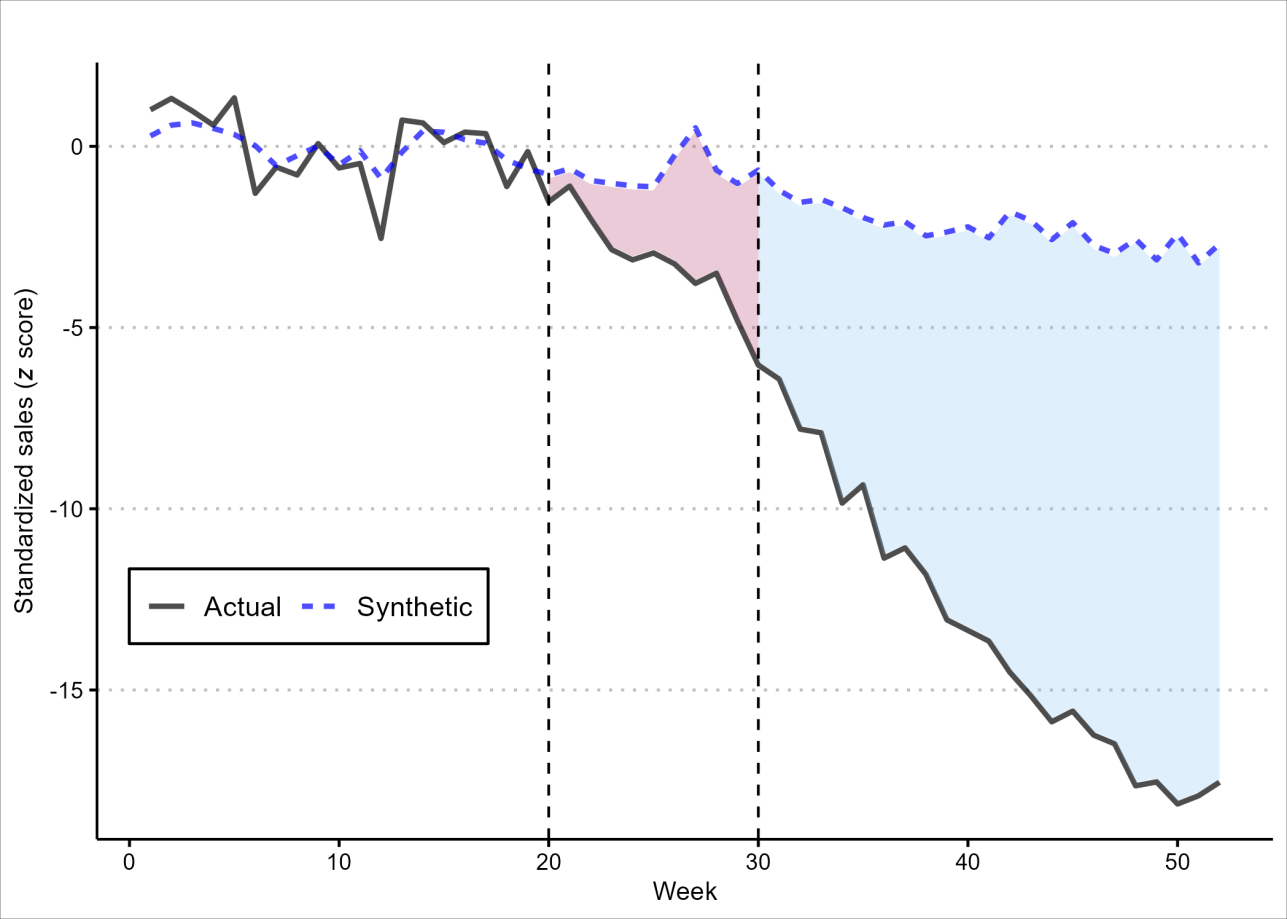
Weekly *z* score standardized unit sales of Esco Bars and the synthetic comparison before and after the US Food and Drug Administration (FDA)’s advisory and enforcement actions. Comparison of weekly observed (actual) versus modeled (synthetic) Esco Bars sales in 2023 in *z* score standardized units [2,3]. The red area represents the difference between the standardized actual and synthetic Esco Bars sales during the intervention period, while the blue area represents the same difference for the postintervention period. Week 1 and week 52 correspond to the week ending January 7, 2023, and the week ending December 30, 2023, respectively. Dashed vertical lines denote the first and last weeks during which the specified FDA advisory and enforcement actions occurred: Week 20 corresponds to the week ending on May 20, 2023, denoting the first week after the FDA’s import alert for Esco Bars was issued on May 12, 2023. Week 30 corresponds to the week ending on July 29, 2023, denoting the week of the FDA’s last advisory action against unauthorized Esco Bars in 2023, warning letters issued to distributors on July 27, 2023.

Figure 1 presents weekly standardized unit sales of Esco Bars and the synthetic comparison in 2023. Unit sales of Esco Bars were very close to that of the comparison, 0.03% lower on average, during the preintervention period. The high similarity provides strong evidence for the parallel trends assumption to support the validity of the SCM model. Our findings also show that Esco Bars sales started to decline the week ending May 21, 2023, immediately after the FDA advisory and enforcement actions, while estimated sales of the synthetic comparison remained largely steady compared to the preintervention period, following a similar moderate downward trend throughout 2023. As a result, during the 5-month postintervention period, between the week ending on July 29, 2023, and the week ending on December 30, 2023, total Esco Bars unit sales were 1.7 million units lower than the synthetic comparison, which represented estimated sales in the absence of FDA intervention. Weekly sales of Esco Bars were 68.5% lower than that of the synthetic comparison by the week ending December 30, 2023.

**Figure 2.**
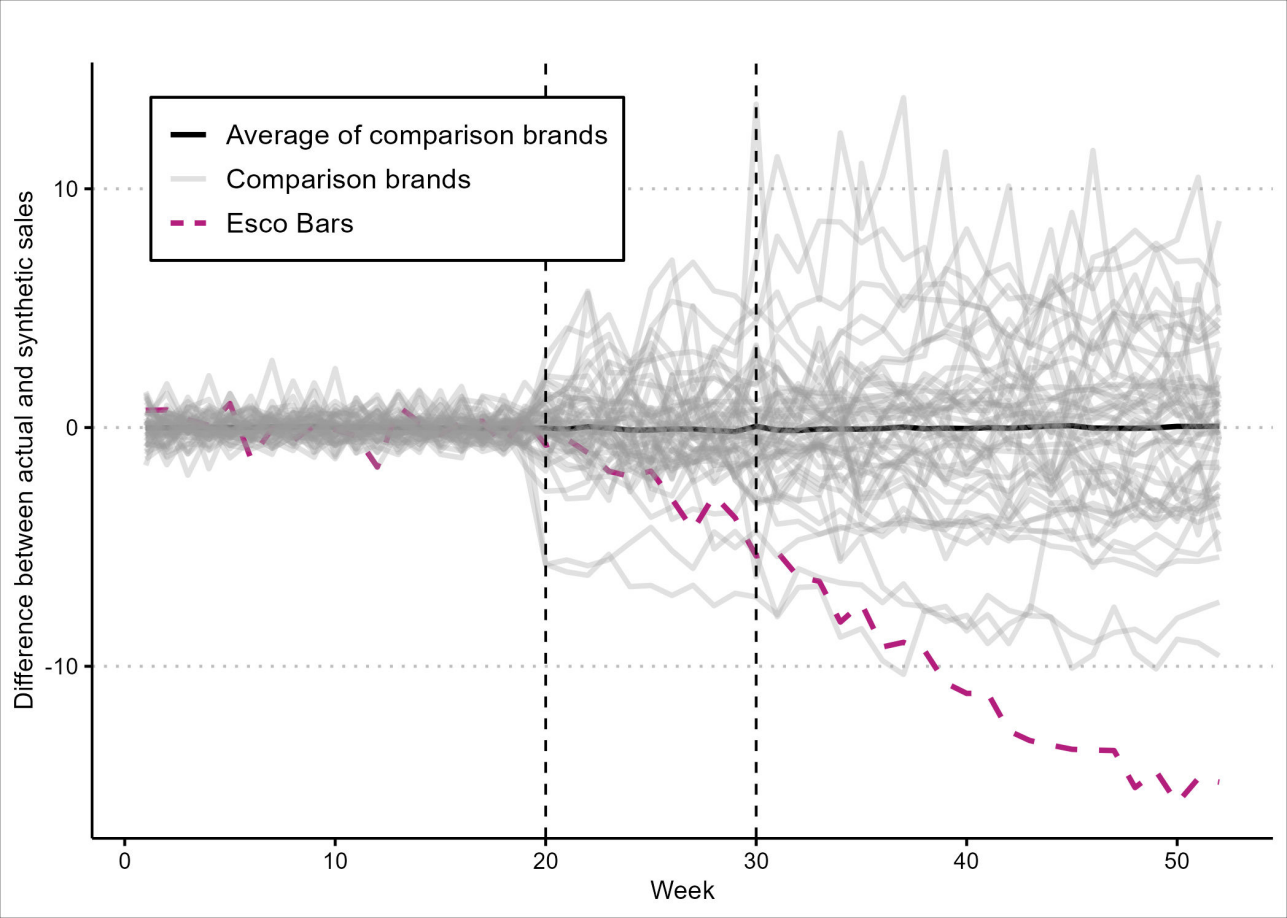
Estimated intervention effect: differences between actual weekly sales for an e-cigarette brand and corresponding synthetic sales. Gray and purple lines represent differences between weekly observed sales for a brand and modeled sales estimated using a corresponding synthetic comparison. Week 1 and week 52 correspond to the week ending January 7, 2023, and the week ending December 30, 2023, respectively. Dashed vertical lines denote the first and last weeks during which the specified US Food and Drug Administration (FDA) advisory and enforcement actions occurred. Week 20 corresponds to the week ending on May 20, 2023, denoting the first week after FDA’s import alert for Esco Bars was issued on May 12, 2023. Week 30 corresponds to the week ending on July 29, 2023, denoting the week of FDA’s last advisory action against unauthorized Esco Bars in 2023, warning letters were issued to distributors on July 27, 2023.

Figure 2 presents the intervention effect of Esco Bars and false intervention effects of all 51 e-cigarette brands in the “donor pool.” In all cases, the effects illustrate the difference between weekly sales of a brand and estimated sales from that brand’s synthetic comparison. Figures 1 and 2 show that Esco Bars sales started to decline immediately following FDA’s first enforcement action, when the import alert for Esco Bars was issued on May 12, 2023. In addition, it also suggests that, compared to all other brands included in the analysis, Esco Bars had the largest sales reduction during the postintervention period (a decline of 14.8 SDs vs 9.5 SDs from Avata, the brand having the second largest sales decline). The findings in Figure 2 further reinforce the causal connection between FDA advisory and enforcement actions subsequent to noncompliance with regulatory review decisions and Esco Bars sales reductions.

**Figure 3.**
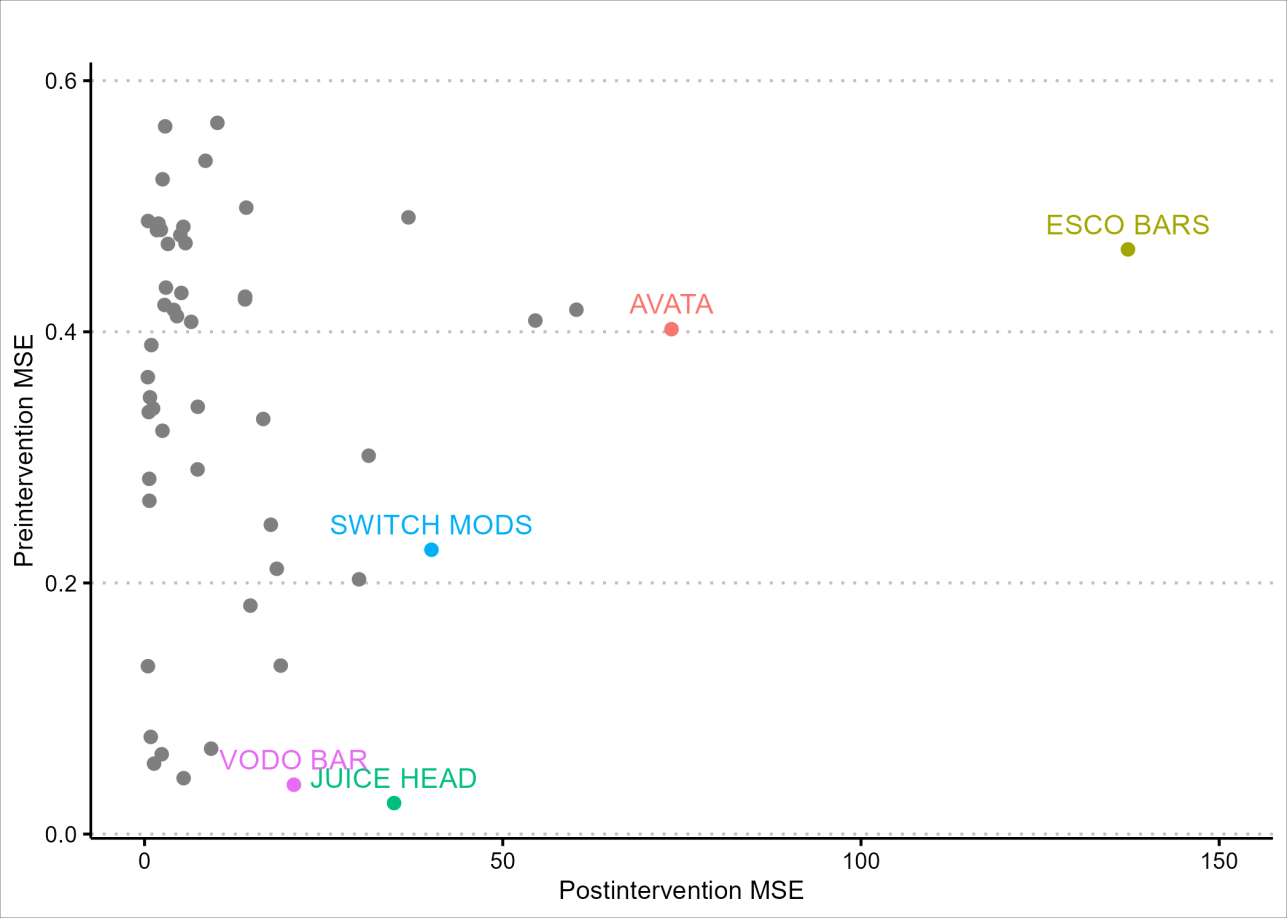
Investigation of esco bars ranking among all e-cigarette brands. Postintervention mean-squared error (MSE) and preintervention MSE were presented for each e-cigarette brand included in this study. MSE measures how close each brand’s actual sales were to modeled sales of the corresponding synthetic comparison. Labels reflect the 5 e-cigarette brands from our sample with the highest ratios of postintervention MSE to preintervention MSE.

We estimated *P* values based on the concept of the Fisher exact test, which depends on the ranking of the ratio of sales changes between the post- and preintervention periods. Esco Bars were ranked third out of the 52 brands in the analysis and thus had a *P* value of .06 (3/52, 5.8%). Two other brands, Juice Head and Vodo Bar, were ranked higher than Esco Bars based on the Fisher exact tests. We further investigated these 2 brands with higher significance (Figure 3). Among all brands included in this analysis, Esco Bars had the largest postintervention MSE by a substantive margin, indicating that in the postintervention period, Esco Bars sales diverged from its synthetic comparison more than any other e-cigarette brand. However, the MSE ratios for Juice Head and Vodo Bar were ranked higher because they had the lowest MSEs in the preintervention period, indicating their sales diverged from their synthetic comparisons less than any other brand in the period. This context suggests that the higher MSE ratios of Juice Head and Vodo Bar do not preclude our finding that the FDA advisory and enforcement actions had significant impacts on Esco Bars sales during the postintervention period.

**Figure 4.**
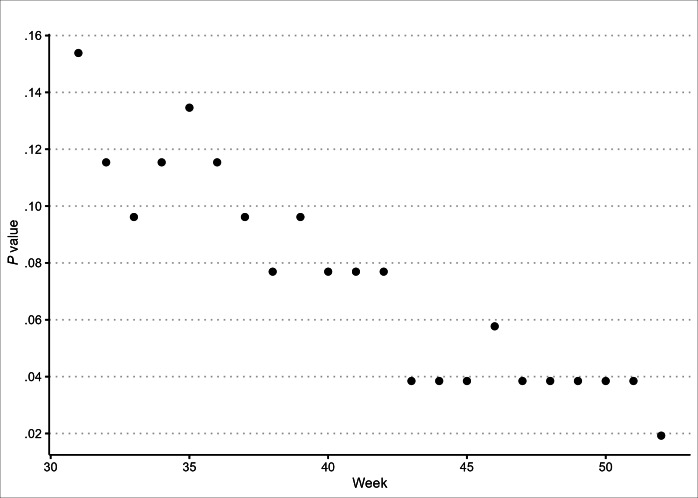
Week-specific *P* values for intervention effect on Esco Bars during the postintervention period. This figure depicts week-specific *P* values for the intervention effect on sales of Esco Bars in the postintervention period in 2023. Week 30 corresponds to the week ending on July 29, 2023, denoting the week of FDA’s last advisory action against unauthorized Esco Bars in 2023, and warning letters were issued to the distributors on July 27, 2023. Week 52 corresponds to the week ending on December 30, 2023, the end of our postintervention period. Following the Fisher exact test, the *P* value for a given week represents the total share of intervention effects (placebo plus actual) that is greater than or equal to the actual intervention effect in a given week. By the final week in the study, the actual intervention effect was the highest out of 52 total intervention effects (1/52, 2%; *P*=.02).

Figure 4 presents *P* values of weekly intervention effects of Esco Bars sales during the postintervention period. In addition to the overall *P* value (.06), week-specific *P* values provide additional evidence on the variations of intervention effects over time. Our findings show that week-specific *P* values decreased rapidly during the postintervention period. The *P* values of the intervention effect immediately after FDA advisory and enforcement actions were .15 (8/52, 15.3%) on the week ending on May 21, 2023, and .02 (1/52, 2%) on the week ending on December 30, 2023. By the 17th week after FDA advisory and enforcement actions, the intervention effect for Esco Bars was statistically significant based on the conventional threshold (*P*<.05). This indicates that these FDA actions were associated with a reduction in Esco Bars sales.

### Sensitivity Analysis

Table 1 presents the results based on alternative data standardization methods. These sensitivity analyses confirmed that our findings are independent of standardization methods for weekly sales. Our major findings remained consistent across all 3 methods, suggesting that the total Esco Bars sales fell by 1.7 million EQ units during the 5 months after the FDA’s advisory and enforcement actions. By the last week of 2023, weekly sales of Esco Bars were reduced by almost 70%, and *P* values of week-specific intervention effects ranged from .02 to .04, reflecting the significant sales changes during the postintervention period.

**Table 1.** Sensitivity analysis results by the standardization method. Three standardization methods were used in this sensitivity analysis: (1) a *z* score method in the main model, which subtracts each brand's preintervention average from its weekly sales and divides the result by its preintervention SD; (2) a min-max normalization method, which subtracts the preintervention minimum and divides by the preintervention range; and (3) a robust rescaling method, which subtracts the preintervention median and divides by the preintervention IQR. The min-max normalization and robust rescaling methods are used for sensitivity analyses.

	*z* score (main model)	Min-max normalization	Robust rescaling
Esco Bars rank among 52 brands	3rd	6th	4th
Overall *P* value	.06	.12	.08
*P* value at the final week in 2023	.02	.04	.04
Total reductions in Esco Bars sales within 5 months (equivalized units)	1.7 million	1.7 million	1.7 million
Weekly Esco Bars sales reduction by the final week of 2023 (%)	68.50	68.50	68.70

## Discussion

### Principal Findings

Our findings demonstrate that the FDA’s advisory and enforcement actions against illicit sales of unauthorized Esco Bars, including the import alert and warning letters that occurred from May to July 2023, resulted in a substantial reduction in product sales within 5 months. These actions not only reduced Esco Bars weekly sales by nearly 70% but also had sustained effects through the end of 2023 in the United States. As a result, total Esco Bars sales were reduced by approximately 1.7 million EQ units over the 5-month period. Notably, our conclusions were consistent and robust across sensitivity analyses.

Our findings confirmed that the FDA’s import alerts and warning letters against Esco Bars products, which were illegally marketed following FDA’s regulatory review decisions, were successful deterrents and had sustained impacts on illicit sales and distribution of these products across the supply chain. It is possible that the manufacturer, distributors, and retailers of these unauthorized products also took voluntary actions to restrain sales and availability of this product after receiving warning letters and/or being placed on import alert. These actions, although voluntary, were under the influence of FDA advisory and enforcement actions, and thus, should be considered as part of the impacts of these actions. These findings reinforce that the FDA’s targeted advisory and enforcement actions are effective against sales and availability of other unauthorized commercial tobacco and nicotine products in the US market.

Our findings also generally align with concurrent patterns of youth cigarette use [7,29]. For example, data from the 2023 and 2024 National Youth Tobacco Survey found substantial declines in youth use of Esco Bars products, dropping from an estimated 440,000 students in 2023 to 160,000 in 2024, with the brand falling out of the top 5 most popular among youth by 2024 [7,29]. Although the focus of these studies was not specifically on FDA advisory and enforcement actions against illicit sales of unauthorized Esco Bars products in 2023, the findings on youth behaviors provide indirect supporting evidence for the effectiveness of these actions.

However, it is also important to note that, although Esco Bars’ sales fell significantly after FDA regulatory review decisions and advisory and enforcement actions, these unauthorized products continued to be available illegally in the United States throughout 2023. This shows that there are actors in the marketplace who are intent on marketing unauthorized products, and thus regulatory vigilance needs to be an active and continual effort.

Accordingly, the FDA has continued to issue additional warning letters and updated import alerts and has pursued enforcement actions against firms to address the continued marketing, distribution, and sales of unauthorized products, including youth-appealing e-cigarettes, beyond 2023. As of October 2024, the FDA had issued more than 700 warning letters to firms for manufacturing, selling, and/or distributing unauthorized new tobacco products including e-cigarettes; issued more than 690 warning letters to retailers, including brick-and-mortar and online retailers, for the sales of unauthorized tobacco products; and filed civil money penalty complaints against more than 75 manufacturers and more than 150 retailers for distribution and/or sales of unauthorized tobacco products [30]. In addition, the Department of Justice (DOJ), in collaboration with the FDA, has filed a total of 8 injunctions against firms that continued to market unauthorized e-cigarette products [31]. Our findings in this analysis suggest that such actions are likely to have similarly successful impacts on regulating the marketplace and enforcing FDA decisions requiring the removal of illegal products from the market.

The complexity of the ever-evolving US e-cigarette marketplace has been exacerbated by the flow of unauthorized e-cigarettes entering the country. The availability and illicit sales of these unauthorized e-cigarette products, many of which are popular among young people, pose serious threats to the health and development of US youth and young adults [3-5,29,32,33]. To address these unauthorized products, the FDA has developed and implemented a comprehensive strategy across the supply chain including with other federal enforcement partners, such as the US Customs and Border Protection and the DOJ.

For example, in collaboration with US Customs and Border Protection, the FDA has made multiple seizures of unauthorized e-cigarettes, including (1) the seizure announced on December 14, 2023, of approximately 1.4 million units of unauthorized e-cigarettes with an estimated retail value of more than US $18 million;[34,35] (2) the seizure announced on April 30, 2024, of more than 45,000 units of unauthorized e-cigarette products with an estimated retail value of US $0.7 million;[36] (3) the seizure announced on May 22, 2025, of nearly 2 million units of unauthorized e-cigarette products with an estimated retail value of US $33.8 million [37]; and (4) the seizure announced on September 10, 2025, of approximately 4.7 million units of unauthorized e-cigarette products with an estimated retail value of US $86.5 million to accelerate further enforcement actions [38]. More recently, as announced on September 25, 2025, the FDA, DOJ, the Bureau of Alcohol, Tobacco, Firearms and Explosives, and the US Marshals Service seized more than 2.1 million unauthorized e-cigarettes taken from 5 distributors and 6 retailers across 7 different states [39].

These enforcement actions are part of the FDA’s comprehensive enforcement strategy across the entire supply chain to increasingly address illicit sales of unauthorized e-cigarette products, particularly for products popular among youth. In addition to these enforcement strategies, the agency continues to educate the public about the risks of these products and to increase voluntary compliance by educating those in the supply chain (eg, retailers) about which products are legal to sell.

Continued surveillance of commercial tobacco and nicotine products available in the United States can provide data on unauthorized product sales and better support FDA efforts to protect the public health, while additional evaluation studies of application review decisions, warning letters, and import alerts can provide further evidence of the effectiveness of the FDA’s regulatory authorities, including product review and advisory and enforcement actions. In addition, given the extent of illicit tobacco product sales globally, including e-cigarette sales, continued surveillance could also help inform regulatory actions across the world, as appropriate.

### Limitations

This study has some limitations. First, the NielsenIQ RMS data did not include e-cigarette sales from Alaska or Hawaii. The data also did not include e-cigarette sales from certain retail outlets, including tobacco specialty stores such as vape shops, liquor stores, and online stores. However, studies based on nationally representative data have shown that less than 25% of current youth e-cigarette users obtained their products from vape shops or online stores [40,41]. Second, this analysis did not account for state or local advisory and enforcement actions against unauthorized e-cigarettes during the analytical time period. However, it is reasonable to assume that these actions would have broadly applied to all unauthorized e-cigarettes and thus were unlikely to differentially affect the estimated impact of this analysis, which was focused on Esco Bars. While this study was not designed to assess the impact of FDA advisory and enforcement actions against unauthorized Esco Bars products on disposable e-cigarette sales, other data have shown that disposable e-cigarette sales have also declined from June 18, 2023, throughout the rest of the year [42]. Third, our findings may underestimate the overall impact of FDA advisory and enforcement actions against Esco Bars, as our analytical period stops at the end of 2023, and the FDA took additional actions in 2024. In addition, since the study was based on retail sales data, we were unable to assess potential behavioral changes among Esco Bars users. However, economic studies have shown that sales and consumption tend to be highly correlated [43-45], and recent studies have shown that Esco Bars has become less popular among youth between 2023 and 2024 [7,29]. Additional research using population-based survey data could present direct behavioral changes associated with the FDA’s tobacco and nicotine product advisory and enforcement actions.

### Conclusions

In response to the illicit sale of Esco Bars e-cigarette products, FDA issued multiple advisory and enforcement actions against firms that marketed, sold, and/or distributed unauthorized tobacco products. Our findings demonstrate that these actions (ie, warning letters and an import alert) substantially reduced sales of Esco Bars products in the United States, with a sustained impact noted over a 5-month period. Specifically, these actions reduced sales by approximately 1.7 million EQ units within 5 months. These findings underscore the importance and impact of FDA actions against unauthorized tobacco products as part of a comprehensive regulatory approach.

## Supplementary material

10.2196/81033 Multimedia Appendix 1 Online appendix: model specifications.

## Abbreviations

DiD: difference-in-difference
DOJ: Department of Justice
FDA: US Food and Drug Administration
MSE: mean squared error
RMS: Retail Measurement Service
RTA: refuse to accept
SCM: synthetic control method
