# Supplementary material for: The Impact of US Food and Drug Administration’s Advisory and Enforcement Actions on US Sales of Esco Bars Products in 2023: Synthetic Control Approach
Source: JMIR Public Health Surveill. 2026 Mar 12;12:e81033. doi: 10.2196/81033 (PMC12981375; doi:10.2196/81033)
Supplement: Multimedia Appendix 1 [file publichealth-v12-e81033-s001.docx]

## ONLINE APPENDIX: MODEL SPECIFICATIONS

### Data Standardization

We standardized EQ unit sales to ensure that the weight for each e-cigarette brand in the donor pool is non-biased, independent of the magnitude of sales value. Otherwise, because of substantial differences in weekly EQ unit sales across brands over the analytical period, the weights of the synthetic comparison are likely to be dominated by a few brands that had relative larger weekly sales, leading to a potentially biased synthetic comparison of Esco Bars sales. Standardizing the sales data enabled us to rescale weekly EQ unit sales of Esco Bars and other 51 e-cigarette brands in the donor pool into a similar range without manipulating actual differences in value.

Z-score standardization is a statistical process converting raw data into z-scores to make them comparable. In this study, we converted the weekly EQ unit sales of each e-cigarette brand $i, i=1,\ldots,52$, into z-scores by subtracting the pre-intervention mean, $\mu_{i,pre}$, from the individual raw weekly sales, $x_{i,t}$, and dividing the difference by the standard deviation in the pre-intervention period , $\sigma_{i,pre}$, as shown in Equation 1.

Equation 1

$$z_{i,t}=\frac{x_{i,t}-\mu_{i,pre}}{\sigma_{i,pre}} for brand i and time t$$

Min-max normalization is another data scaling technique used in the sensitivity analysis. This approach transforms raw data into a common scale, between 0 to 1. To rescale the NIQ weekly sales data, we subtracted the minimum sales value in the pre-intervention period from the individual weekly sales and then divided the difference by the difference between the maximum and minimum sales value in the pre-intervention period for each e-cigarette brand.

Equation 2

$${mm}_{i,t}=\frac{x_{i,t}-{\min(x}_{i,pre})}{max\left( x_{i,pre} \right)-{\min(x}_{i,pre})} for brand i and time t$$

We also tested the robustness of our findings by applying robust scaling to normalize weekly EQ unit sales. The robust scaling uses the median and interquartile range (IQR) to rescale data. We subtracted the median sales in the pre-intervention period from the individual weekly sales and divided the difference by the interquartile range of the individual weekly sales in the pre-intervention period for Esco Bars and each e-cigarette brand in the donor pool.

Equation 3

$${rs}_{i,t}=\frac{x_{i,t}-{\mathrm{median}(x}_{i,pre})}{{\mathrm{IQR}(x}_{i,pre})} for brand i and time t$$

### Model specification: A difference-in-differences model with a synthetic control method

Let $y_{EB, t}$be rescaled Esco Bars EQ unit sales and $y_{c, t}$be that of each of the 51 e-cigarette brands, $c=1,\ldots,C$, in the donor pool for time periods $t=1,\ldots,T$. Let $T_{0}$ be the number of time periods before FDA advisory and enforcement actions. Assume that all weights are non-negative, as sales cannot go below 0, and that the sum of all weights equals 1. The difference in rescaled sales between Esco Bars and the synthetic comparison in time t, $\hat{y}_{EB, t}, for t>T_{0}$, can be expressed by the equation below.

Equation 4

$${\hat{y}_{EB,t}=y}_{EB,t}-\sum_{c=1}^{C} w_{c}y_{c,t} for t>T_{0}, where w_{c}\geq0 and \sum_{c=1}^{C} w_{c}=1$$

assuming

$$\frac{dy_{EB,t}}{dt}-\frac{d\sum_{c=1}^{C} w_{c}y_{c,t}}{dt}\approx0 for t\leq T_{0}$$

$\hat{y}_{EB}=\sum_{t>T_{0}}^{T} \hat{y}_{EB, t}$, the cumulative difference between the rescaled sales of Esco Bars and that of synthetic comparison in the post-intervention period, represents the impact of FDA advisory and enforcement actions on Esco Bar sales. However, the synthetic control method (SCM) does not provide a conventional approach to estimate the statistical significance of the outcome, $\hat{y}_{EB}$. Therefore, following established techniques for the SCM[1], we measured *P* value following the concept of Fisher’s Exact Test.[2] This test allows us to measure false intervention effects, which estimate individual sales changes for each e-cigarette brand in the donor pool as if FDA advisory and enforcement actions took place against that e-cigarette brand while other conditions remain constant. Then, we compared false intervention effects to the intervention effect of Esco Bars to assess if the latter is statistically significant.

To conduct Fisher’s Exact Test, we first created synthetic comparisons for each e-cigarette brand in the donor pool. Second, for each of the cigarette brands, including Esco Bars, we measured the difference between actual and estimated sales of its own synthetic comparison in the post-intervention period as shown in Equation 4. Then, we calculated the average of the squared discrepancies between actual and estimated sales of its synthetic comparison in the post-intervention period, i.e., mean squared error (MSE) for each brand $i, i=1,\ldots,52$, as shown in Equation 5.

Equation 5

$${MSE}_{i, t>T_{0}}=\frac{1}{\sum_{t+1}^{T_{0}} 1}(y_{i,t}{-\sum_{i=1}^{N} w_{i}y_{i,t})}^{2} for t>T_{0} and i=1,\ldots,52$$

Then, we normalized the post-intervention MSE by the pre-intervention MSE for each brand *i*, i.e., taking the ratio of the post-intervention MSE to the pre-intervention MSE to account for variations in difference between actual and estimated sales of the synthetic comparison in the pre-intervention period as shown in Equation 6.[3] Through this process, the ratio of the post-intervention MSE to the pre-intervention MSE can represent the comparable magnitude of the intervention effect of Esco Bars and the false intervention effect of other e-cigarette brands in the post-intervention period, respectively.

Equation 6

$$\hat{r}_{i}=\frac{{MSE}_{i, t\leq T_{0}}}{{MSE}_{i,{t>T}_{0}}} for i=1,\ldots,52$$

Finally, we compared the relative magnitude of false intervention effects, $\hat{r}_{c},$ of each e-cigarette brand to that of the intervention effect on Esco Bars, $\hat{r}_{EB}$, and counted the number of false intervention effects that are greater than or equal to the intervention effect. As a result, the *P* value can be measured as the proportion of false intervention effects that are greater than or equal to the intervention effect as shown in Equation 7:

Equation 7

$$P=\frac{1}{N}\sum_{i=1}^{N} 1(\hat{r}_{i}\geq\hat{r}_{EB,}) for i=1,\ldots,52$$

The impact of the FDA advisory and enforcement actions on Esco Bars sales is statistically meaningful if *P* value is sufficiently small.

**Appendix Table 1. E-cigarette brand weights for the synthetic Esco Bars**

| **Brand** | **Ten brands with non-zero weights in the synthetic comparison** |
| --- | --- |
| Esco Bars | ZEO (30.6%), Sigma E Hookah (17.4%), Glamee Nova (14.9%), Youup (12.4%), Clean Cig (11%), POP (4.6%), Cig2o (3.5%), UNO (2.7%), Logic (2.2%), Air Bar (0.6%) |

Notes: Other e-cigarette brands in the donor pool with zero weights include: Airis, Alphaa, Avata, Bidi Stick, Bloko, Bud Vape, Bummy, Cloud, E S, Epic, Ezzy, Flum, Fruty Stik, Glas, Green Smart Living, Ignite, Jak, Juice Head, Juul, Kado Bar Leap, Luto, Mr Fog, Myle, Njoy, Omega Plus, Orion, Puur Vapor, R&M Switch, Randm Tornado, Space Ultra M5, Square, Stagbar, Stig, Switch Mods, Tkca Squid 5k, Tugpod, Uno Bar, Vodo Bar, Yme, Z Stick.

**Appendix Figure 1. Weekly Z-Score Standardized Unit Sales of Esco Bars and the Synthetic Comparison Before and After FDA Initial Advisory and Enforcement Action**

**
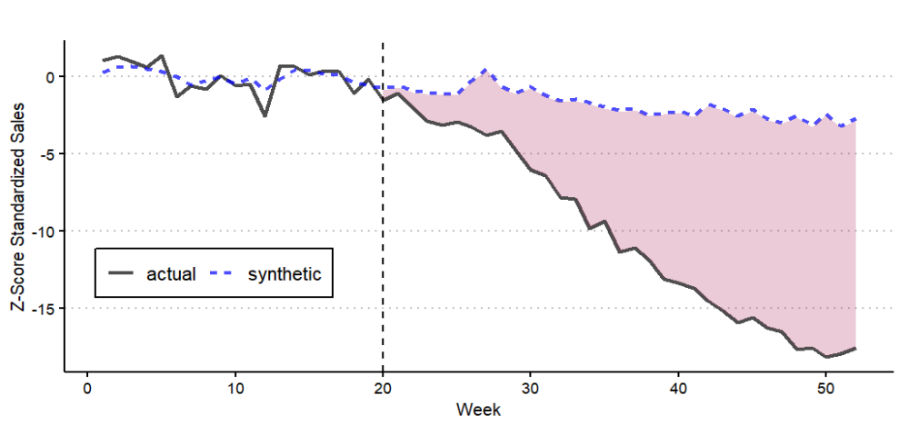
**

Notes: Comparison of weekly observed (actual) vs modeled (synthetic) Esco Bars sales in 2023 in Z-score standardized units. Week 1 and week 52 correspond to the week ending January 7, 2023 and the week ending December 30, 2023, respectively. Dashed vertical lines denote the first and last weeks during which the specified FDA enforcement actions occurred: week 20 corresponds to the week ending May 20, 2023, denoting the first week after FDA’s import alert for Esco Bars issued on May 12, 2023. Week 30 corresponds to the week ending July 29, 2023, denoting the week of FDA’s last advisory action against unauthorized Esco Bars in 2023, warning letters issued to distributors on July 27, 2023.

**Appendix** **Figure 1** presents weekly unit sales of Esco Bars and the synthetic comparison in 2023, immediately after FDA’s initial enforcement action of import alert. Our results show, between the week ending on May 20^th^ and the week ending on December 30^th^, total Esco Bars sales were 1.9 million EQ units lower than the synthetic comparison, which indicates that additional 0.2 million EQ unit sales (1.9-1.7 million) were reduced during the intervention period.

## APPENDIX REFERENCES

1. Abadie A, Diamond A, Hainmueller J. Comparative Politics and the Synthetic Control Method. American Journal of Political Science. 2015;59(2):495-510. doi: <https://doi.org/10.1111/ajps.12116>.

2. Graham JGU. Fisher's Exact Test. Journal of the Royal Statistical Society: Series A (Statistics in Society). 1992;155(3):395-402. doi: 10.2307/2982890.

3. Abadie A, Diamond A, Hainmueller J. Synthetic Control Methods for Comparative Case Studies: Estimating the Effect of California’s Tobacco Control Program. Journal of the American Statistical Association. 2010;105(490):493-505. doi: 10.1198/jasa.2009.ap08746.
